# Systems Biology of Gut Microbiota-Human Receptor Interactions: Toward Anti-inflammatory Probiotics

**DOI:** 10.3389/fmicb.2022.846555

**Published:** 2022-03-03

**Authors:** Lokanand Koduru, Meiyappan Lakshmanan, Shawn Hoon, Dong-Yup Lee, Yuan Kun Lee, Dave Siak-Wei Ow

**Affiliations:** ^1^Institute of Molecular and Cell Biology, Agency for Science, Technology and Research (A*STAR), Singapore, Singapore; ^2^Bioprocessing Technology Institute, Agency for Science, Technology and Research (A*STAR), Singapore, Singapore; ^3^Bioinformatics Institute, Agency for Science, Technology and Research (A*STAR), Singapore, Singapore; ^4^School of Chemical Engineering, Sungkyunkwan University, Suwon, South Korea; ^5^Department of Microbiology and Immunology, Yong Loo Lin School of Medicine, National University of Singapore, Singapore, Singapore; ^6^Department of Surgery, Yong Loo Lin School of Medicine, National University of Singapore, Singapore, Singapore

**Keywords:** systems biology, gut microbiota, inflammatory disorders, probiotics, postbiotics

## Abstract

The incidence and prevalence of inflammatory disorders have increased globally, and is projected to double in the next decade. Gut microbiome-based therapeutics have shown promise in ameliorating chronic inflammation. However, they are largely experimental, context- or strain-dependent and lack a clear mechanistic basis. This hinders precision probiotics and poses significant risk, especially to individuals with pre-existing conditions. Molecules secreted by gut microbiota act as ligands to several health-relevant receptors expressed in human gut, such as the G-protein coupled receptors (GPCRs), Toll-like receptor 4 (TLR4), pregnane X receptor (PXR), and aryl hydrocarbon receptor (AhR). Among these, the human AhR expressed in different tissues exhibits anti-inflammatory effects and shows activity against a wide range of ligands produced by gut bacteria. However, different AhR ligands induce varying host responses and signaling in a tissue/organ-specific manner, which remain mostly unknown. The emerging systems biology paradigm, with its powerful *in silico* tool repertoire, provides opportunities for comprehensive and high-throughput strain characterization. In particular, combining metabolic models with machine learning tools can be useful to delineate tissue and ligand-specific signaling and thus their causal mechanisms in disease and health. The knowledge of such a mechanistic basis is indispensable to account for strain heterogeneity and actualize precision probiotics.

## Introduction

The human gut microbiome has attracted attention in the past decade for its role in inflammatory disorders (Kamada et al., 2013). Altering the gut microbiome profile through probiotic supplementation has been suggested as a potential strategy for ameliorating the symptoms associated with such diseases including inflammatory bowel disease (IBD) and colorectal cancer (Hendler and Zhang, 2018). The commercial probiotic formulations largely exist as generic supplements labeled with health claims that often remain unsubstantiated. Moreover, the existence of non-specific labels affects public perception on their proposed efficacy in treating health conditions, thereby posing risk to the probiotic market over a long run. Hence, precision probiotics with well-defined activities that target specific health conditions are imperative. The development of precision probiotics is not a trivial task, as it requires a deeper understanding of the complex molecular cross talk between gut microbiota and host. One way to address this problem is to first map possible interactions between the two in the gut environment and subsequently trace the host effects downstream to the interaction. Several human receptors have been shown to interact with the gut microbiota, including the G-protein coupled receptors (GPCRs), Toll-like receptors (TLRs), pregnane X receptor (PXR), and aryl hydrocarbon receptor (AhR) (Mohandas and Vairappan, 2017; Chang and Kao, 2019; Chen et al., 2019). The activity of each of these receptors brings about varied molecular responses that affect health. In simpler terms, the “precision” aspect of probiotics can be gauged by their net conceivable interactions with the host receptors.

Tracing the physiological effects of gut microbiota, particularly during probiotic supplementation, and the small molecules they produce is a daunting task. In particular, the pleiotropy resulting from the interaction of multiple gut microbial ligand-host receptor combinations is hard to discern using classical molecular biology techniques. Integration of multiple omics datasets has been instrumental in explaining complex biological phenomena. Further, genome-scale metabolic models (GEMs) serve as convenient tools to represent complex molecular networks in a meaningful manner, especially when constrained using omics datasets. Therefore, the future of gut microbiome therapeutics will greatly benefit from a systematic amalgamation of multiomics, GEMs and machine learning tools.

## Gut Microbiota-Human Receptor Interactions

Gut microbiota interact with the host either through direct association with the intestinal epithelial cells or *via* secretion of small molecules. Although direct interactions are possible, they are limited to human cells in the gut lining. On the other hand, small molecules produced by the microbiota in gut environment not only interact with the host cell lining in intestine but also enter the blood circulation and access receptors expressed in nearly all human organs. Such interactions play a wide array of roles in health and disease, ranging from intestinal homeostasis, dysbiosis, inflammation and cancer. The human receptors such as GPCRs, PXR, and TLRs have been shown to play an important role in gut-microbiome mediated immunomodulation including their anti-inflammatory roles (Valentini et al., 2014; Venkatesh et al., 2014; Chen et al., 2019; Melhem et al., 2019). However, AhR is more often implicated in governing the overall gut homeostasis and inflammation (Zelante et al., 2013; Marinelli et al., 2019). Several studies have clearly demonstrated causal links between anti-inflammatory outcomes in chronic inflammatory conditions and treatment with AhR ligands or the gut microbial strains synthesizing them (Takamura et al., 2011; Fukumoto et al., 2014; Lamas et al., 2016; Abdulla et al., 2021; Zhao et al., 2021). AhR is a transcription factor, originally thought to function as a xenobiotic sensor, is now known to be an important regulator of immunity, stem cell maintenance and cellular differentiation (Gutiérrez-Vázquez and Quintana, 2018). Several signaling mechanisms downstream to AhR activation have been elucidated (Agus et al., 2018; Roager and Licht, 2018). Different gut microbial species and strains exhibit varied AhR modulating capabilities *via* their secretory metabolites. For example, short chain fatty acids (SCFAs) produced by gut microbiota bring about AhR activity through multiple mechanisms, including enhancement of AhR responsiveness to ligands (Jin et al., 2017), acting as a direct ligand to AhR (Marinelli et al., 2019), and increasing the expression of AhR (Yang et al., 2020). Each of these studies have indicated that the SCFA-mediated mechanisms eventually converge at promoting AhR activity and bring about anti-inflammatory effects. Similarly, tryptophan and indole derivatives activate the AhR resulting in anti-inflammatory outcomes (Lamas et al., 2016; Özçam et al., 2019). Despite their promise, the ligand- and tissue-specific effects of AhR signaling, which confound clinical studies, is not completely understood; not all AhR ligands confer anti-inflammatory phenotype (Denison et al., 2011). Therefore, characterizing strains and their tissue-specific AhR-mediated inflammation is an indispensable requirement for their use as therapeutic modalities.

## Role of Systems Biology

Gut is a complex environment characterized by innumerable biotic and abiotic interactions. The chaotic regimen consisting of diet, microbiota, host secretions and gut barrier render the ligand-receptor interactions largely untraceable using conventional “top-down” experimental techniques (Veiga et al., 2020). However, the emergent network properties of such systems of interacting components have always been tractable using mathematical modeling techniques. Systems biology, a discipline that treats organisms as an integration of one or more such networks, plays an important role in today’s biological research, especially studying complex systems such as the human gut. An aspect that makes a strong case for the use of systems biology has been its tremendous success in studying metabolic networks using constraint-based modeling approach. Large-scale metabolic model reconstruction efforts have been undertaken in the past to facilitate studies deciphering interactions within and between gut microbiota and human intestinal cells (Diener et al., 2020; Heinken et al., 2021). These models largely rely on biochemical capabilities reflected by genomic data and taxonomic abundances estimated from metagenomic information, and therefore, the depth of interactions prevailing in the gut extend beyond what is usually captured by them. Fecal and serum metabolomic and transcriptomic datasets when integrated with such metabolic models can systematically reveal metabolic correlations of the microbiome. Furthermore, the use of machine learning tools in combination with omics integrated metabolic models can help derive mechanistic insights on potential health effects of specific strains (Yang et al., 2019; Lewis and Kemp, 2021). A list of publicly available systems biology tools that can aid in such an integrated analysis is shown in Table 1.

**TABLE 1:** Survey of systems biology tools and resources to study gut microbiome-host receptor interactions.

Name of the tool/resource	URL	References
**Ligand-receptor pairs in humans and mice**		
Compiled by Lewis Lab at UCSD	https://github.com/LewisLabUCSD/Ligand-Receptor-Pairs	Armingol et al., 2021
CellTalkDB	http://tcm.zju.edu.cn/celltalkdb/	Shao et al., 2021
**Database of available microbiome and host genome-scale models**		
BiGGModels	http://bigg.ucsd.edu/	Norsigian et al., 2020
BioModels	https://www.ebi.ac.uk/biomodels/	Malik-Sheriff et al., 2020
AGORA	https://www.vmh.life/	Magnúsdóttir et al., 2017
Human model (Recon)	https://www.vmh.life/	Noronha et al., 2019
metaGEM	https://github.com/franciscozorrilla/metaGEM	Zorrilla et al., 2021
Metabolic Atlas	https://metabolicatlas.org/	Wang et al., 2021
**Tools to reconstruct microbiome and host genome-scale models**		
RAVEN Toolbox	https://github.com/SysBioChalmers/RAVEN	Wang et al., 2018
COBRA toolbox/COBRAPy	http://opencobra.github.io/	Heirendt et al., 2019
gapSeq	https://github.com/jotech/gapseq	Zimmermann et al., 2021
CarveMe	https://carveme.readthedocs.io/en/latest/index.html	Machado et al., 2018
ModelSEED	https://modelseed.org/	Seaver et al., 2021
Memote	https://memote.io/	Lieven et al., 2020
**Tools to simulate genome-scale metabolism of microbiome and hosts and their interactions**		
RAVEN Toolbox	https://github.com/SysBioChalmers/RAVEN	Wang et al., 2018
COBRA toolbox/COBRAPy	http://opencobra.github.io/	Heirendt et al., 2019
MICOM	https://github.com/micom-dev/micom	Diener et al., 2020
CASINO		Shoaie et al., 2015
SteadyCom	https://github.com/maranasgroup/SteadyCom	Chan et al., 2017
COMETS	https://github.com/segrelab/comets	Harcombe et al., 2014
CODY	https://github.com/JunGeng-Sysbio-Chalmers/CODY1.0_SourceCode	Geng et al., 2021

## Precision Anti-Inflammatory Probiotics: Need for a Two-Pronged Approach

The current trend in systems biology has been to predict and quantify immunomodulatory effects of specific probiotic strains and metabolites that they produce in the gut environment (dos Santos et al., 2010; Fiocchi and Iliopoulos, 2021). Availability of quantitative information on microbiota-induced immunomodulation is an essential requirement to realize precision probiotics. Ligand-receptor interactions form the direct link between microbiota and immunomodulation in humans. Therefore, a systematic approach toward designing precision probiotics must first take into account the sum total interactions brought about by each bacterial strain or microbial consortia *via* the small molecule ligands they secrete and their possible receptors in humans. In this regard, GEMs can greatly simplify the evaluation of various strains for the biosynthesis of potential ligands. Well-curated models can further provide quantitative estimates of the ligand production, which may serve as proxies for ligand-receptor activities. For instance, we have reconstructed and used GEMs for 18 lactic acid bacterial strains to systematically evaluate their probiotic capabilities (Koduru et al., 2021). This study constitutes one of the first attempts to theoretically quantify the probiotic potential of strains. Specifically, we relied on three important metrics based on constraint-based flux analysis to evaluate the strains. First, we estimated their capacities to biosynthesize metabolites, commonly referred to as “postbiotics,” with anti-inflammatory properties such as SCFAs, tryptophan and indole, and pro-inflammatory metabolites such as lipoteichoic acids and branched chain amino acids. Subsequently, we evaluated their ability to persist in the gut environment under various dietary regimens. Finally, we assessed the nature of their potential interactions with beneficial gut commensals. These estimates can be prudently used to recommend strains to manage inflammation.

**FIGURE 1:**
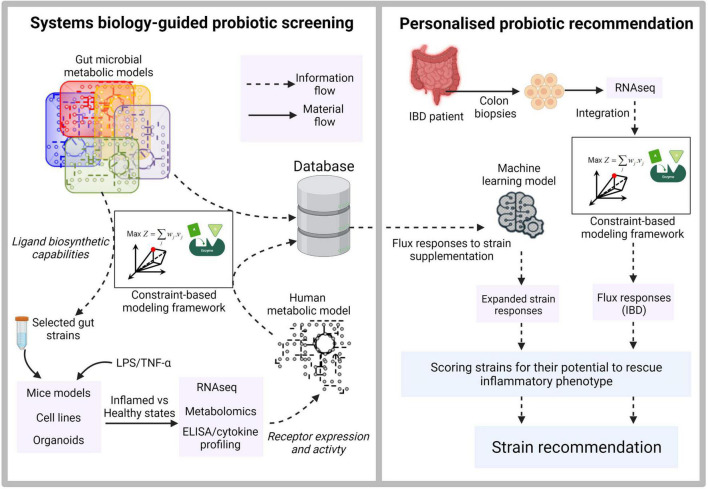
Illustration of systems biology-guided personalized probiotic recommendation. The first stage consists of constraint-based model-driven screening of gut microbial strains for the production of diverse ligands capable of binding to human receptors. The secretory metabolites from selected strains are then used to treat mice models, cell lines or organoids. Their inflamed and healthy states are quantified using inflammatory cytokine profiling. Paired RNAseq and metabolomic datasets derived from each probiotic supplementation scenario provide information on the expression and activity of the target receptors and serve as constraints to derive context-specific models from a generic human genome-scale metabolic network. The context-specific models are used to derive flux responses corresponding to the supplementation of each probiotic strain. The second stage involves training a machine learning model using the extensive flux response information derived in the first stage. RNAseq data derived from real IBD patients for whom the probiotic recommendations are to be made is used to generate a context-specific model and the corresponding metabolic flux distribution. This flux distribution is then used as an input to the trained machine learning model to rank strains based on their ability to rescue the inflammatory phenotype.

The second important requirement of precision probiotics involves understanding the nature of pleiotropic effects resulting from the cumulative downstream signaling of multiple ligand-receptor interactions. To this end, (Stein et al., 2018) have developed a computational approach to design optimal microbial consortia for induction of regulatory T-cells (Tregs), which are immune cells with an anti-inflammatory effect. The authors combined data on the contribution of 11 Clostridia strains in Treg induction with an ecological model to identify and rank different stable combinations for their ability to promote Treg activation. Importantly, they introduce and validate a computational metric called Treg Induction Score (TrIS) to facilitate the systematic selection of immunomodulatory strains. Interestingly, at least part of AhR’s anti-inflammatory effects can be attributed to its *in vivo* role as a potent Treg inducer (Goettel et al., 2016; Ye et al., 2017), an angle that was not pursued by Stein et al. (2018). In this regard, characterization of each of the 11 Clostridia strains for their potential to synthesize AhR ligands could further provide a thorough mechanistic basis of their Treg inducing capabilities, as well as, potentially help fine-tuning the microbial consortia for optimal activity. Therefore, while such studies constitute big steps in the direction of precision probiotics, integrating them with metabolic models and machine learning tools can yield insights into metabolites beyond the extensively investigated SCFAs, potentially paving ways to decipher the functional aspects of “large metabolic unknowns” of the gut microbial world. Furthermore, the precision aspect can be strengthened by gaining individual-specific understanding of the host physiology. An illustration of a systematic approach to integrate multiple omics datasets, metabolic models and machine learning to screen and make personalized probiotic recommendations targeting IBD is shown in Figure 1. Although the focus here is on the microbiome-host interactions in gut environment, a similar approach can be extended to such interactions in skin, oral and vaginal microbiome.

## Conclusion

The conventional top-down approach of probiotic discovery largely relies on correlations between microbiota composition in gut or fermentative food and health or disease (Veiga et al., 2020). Although this approach has been successful in recommending candidate strains to promote gut health, the absence of a mechanistic basis limits their application to “generic” probiotic supplements. Inflammatory disorders such as IBD and colorectal cancer, being complex manifestations of unhealthy gut and microbiota dysbiosis extensively depend on host-genetic, age, diet and other environmental factors, require precision probiotic targeting. In addition, although gut microbial metabolites such as the AhR ligands have shown tremendous potential in ameliorating inflammation, their exact role might vary depending on the cells, tissue and organs expressing AhR (Cannon et al., 2022). The bottom-up approach guided by systems biology proposed here involves a two-pronged strategy – comprehensive characterization of receptor activating potential of strains and cell/tissue-specific pleiotropic signaling effects downstream to receptor activation in humans. We reason that the future transition toward precision probiotics thus lies in deciphering ligand-receptor interactions, with AhR being a key mediator in managing chronic inflammation.

## Data Availability Statement

The original contributions presented in the study are included in the article/supplementary material, further inquiries can be directed to the corresponding authors.

## Author Contributions

LK drafted the manuscript. ML, SH, D-YL, YL, and DO edited and proof-read the manuscript. All authors made a substantial contribution to the work and approved it for publication.

## Conflict of Interest

The authors declare that the research was conducted in the absence of any commercial or financial relationships that could be construed as a potential conflict of interest.

## Publisher’s Note

All claims expressed in this article are solely those of the authors and do not necessarily represent those of their affiliated organizations, or those of the publisher, the editors and the reviewers. Any product that may be evaluated in this article, or claim that may be made by its manufacturer, is not guaranteed or endorsed by the publisher.
